# Altering neuronal excitability to preserve network connectivity in a computational model of Alzheimer's disease

**DOI:** 10.1371/journal.pcbi.1005707

**Published:** 2017-09-22

**Authors:** Willem de Haan, Elisabeth C. W. van Straaten, Alida A. Gouw, Cornelis J. Stam

**Affiliations:** 1 Department of Clinical Neurophysiology and MEG, VUmc, Amsterdam, The Netherlands; 2 Alzheimer Center and Department of Neurology, VUmc, Neuroscience Campus Amsterdam, Amsterdam, The Netherlands; Université Paris Descartes, Centre National de la Recherche Scientifique, FRANCE

## Abstract

Neuronal hyperactivity and hyperexcitability of the cerebral cortex and hippocampal region is an increasingly observed phenomenon in preclinical Alzheimer’s disease (AD). In later stages, oscillatory slowing and loss of functional connectivity are ubiquitous. Recent evidence suggests that neuronal dynamics have a prominent role in AD pathophysiology, making it a potentially interesting therapeutic target. However, although neuronal activity can be manipulated by various (non-)pharmacological means, intervening in a highly integrated system that depends on complex dynamics can produce counterintuitive and adverse effects. Computational dynamic network modeling may serve as a virtual test ground for developing effective interventions. To explore this approach, a previously introduced large-scale neural mass network with human brain topology was used to simulate the temporal evolution of AD-like, activity-dependent network degeneration. In addition, six defense strategies that either enhanced or diminished neuronal excitability were tested against the degeneration process, targeting excitatory and inhibitory neurons combined or separately. Outcome measures described oscillatory, connectivity and topological features of the damaged networks. Over time, the various interventions produced diverse large-scale network effects. Contrary to our hypothesis, the most successful strategy was a selective stimulation of all excitatory neurons in the network; it substantially prolonged the preservation of network integrity. The results of this study imply that functional network damage due to pathological neuronal activity can be opposed by targeted adjustment of neuronal excitability levels. The present approach may help to explore therapeutic effects aimed at preserving or restoring neuronal network integrity and contribute to better-informed intervention choices in future clinical trials in AD.

## Author summary

Alzheimer’s disease (AD) is a growing burden on society, without a cure in sight. Pathological high neuronal activity and excitability is an increasingly observed phenomenon in early stage AD. Its exact role in the disease process is unclear, but it may form an interesting therapeutic target. However, although brain dynamics can be influenced in many ways, the highly complex nature of the brain makes it difficult to predict what approach will be most effective. To test our hypothesis that neuronal hyperactivity can be countered effectively by altering neuronal excitability levels, we examined various strategies aimed at preserving brain network integrity in a computational AD model of the human brain. Of these strategies, a scenario involving stimulation of excitatory neurons extends the period with normal network function most successfully. The results of this ‘virtual trial’ suggest that network effects of pathological neuronal activity can be opposed by selective altering of neuronal excitability levels. In general, this approach can explore therapeutic effects aimed at preserving or restoring brain network integrity, and thereby contribute to selecting promising interventions for future clinical trials in AD.

## Introduction

Electrophysiologically, Alzheimer’s disease (AD) is commonly characterized by ‘negative’ findings: a gradual, diffuse slowing of brain activity (notably the posterior dominant rhythm), decreases in functional connectivity, and a loss of network structure and complexity [1–3]. In recent years however, various studies have reported observations that challenge this notion: in the preclinical AD, Mild Cognitive Impairment (MCI) and early AD stages, neuronal hyperactivity and increased functional connectivity has been observed at various scales [4–11]. While these increases were first interpreted as a compensation mechanism for synaptic dysfunction in AD, the evidence now clearly points in a different direction; neuronal hyperactivity as a part of the pathophysiological cascade in AD. For instance, it has been shown that damage induced by the typical amyloid deposits in AD leads to neuronal hyperexcitability and disinhibition [12,13]. In turn, neuronal hyperactivity *itself* has also been demonstrated to drive amyloid deposition rates [14,15], a very intriguing finding that has led some to hypothesize that neuronal dynamics may play a causal role in AD pathophysiology, possibly as part of a positive feedback loop involving the neurotoxic amyloid deposits [16–18]. Additional, indirect support for this view is provided by the very early phase in which neuronal dynamics and connectivity are disrupted in AD, the independent associations of AD risk factors (age and ApoE4 status) with neuronal activity levels, and the increased incidence of epilepsy and epileptiform neuronal activity in this population [6,9,19–27].

Regardless of the exact role of disrupted neuronal dynamics in the pathological cascade of AD, the observations described above may have therapeutic relevance, since neuronal or synaptic function can be targeted effectively by various (non-) pharmacological means. In fact, the current medicinal (symptomatic) treatment for AD aims to improve neuronal communication by enhancing cholinergic or glutamatergic neurotransmission [28–30]. Unfortunately, the large majority of the current clinical trials are aimed at decreasing amyloid load, while investigations on improving neuronal function are relatively limited. Nevertheless, a few recent studies have confirmed that targeting neuronal or synaptic behavior may be beneficial in AD. For example, experimental studies in rats and in humans with mild cognitive impairment (MCI) indicated that the anti-epileptic drug levetiracetam diminishes hippocampal hyperactivity while improving cognitive performance [31]. Also, enhancement of synapse formation and function by medical nutrition was reported to have a positive effect on memory function in AD patients [32,33]. Non-pharmacological therapeutic studies with the specific aim to modify neuronal activity (e.g. deep brain stimulation or transcranial magnetic stimulation) have not yet produced significant clinical benefits in AD, but are under development [34–36].

With these possibilities in mind, a key question is how to predict and optimize the effect of therapy in the complex, highly interconnected and highly dynamic human brain. While therapeutic strategies usually target specific brain areas or structures, it is naïve to expect that local interference will not influence surrounding or connected parts of the brain. As our awareness of the brain as a complex, distributed system is growing, we should appreciate the consequences: even subtle changes can have wide-ranging and paradoxical effects [37,38]. Variations in efficacy and unforeseen or adverse effects are frequently encountered in neurological therapy, and perhaps could be partly explained by our lack of insight in this regard. How can we get a grip on this complex behavior, in order to guide our hypotheses and experiments more reliably?

In recent years, studies of the complexity of the brain have entered a new era, due to rapid advances in data acquisition technology and the powerful application of theoretical concepts of complex network analysis [39,40]. In clinically oriented studies, this method has mainly been employed to interpret patient data in order to gain a better understanding of disease mechanisms or to find diagnostic and prognostic markers of disease [41,42]. However, top-down patient-driven research is not its sole application: the combination of network analysis and computational modeling can offer an interesting complementary bottom-up approach [43–45]. For example, several studies have combined neural mass modeling with network analysis to study the effect of lesions on the brain [38,46]. With regard to AD, a previous study from our group indicated that neuronal hyperactivity may play a substantial role in the disease mechanism by demonstrating that an activity-dependent degeneration regime yielded remarkably similar results to studies in AD and mild cognitive impairment (MCI) patients, including selective hub vulnerability and initial neuronal hyperactivity followed by slowing, disconnection, and loss of network topology [18,47].

**Fig 1:**
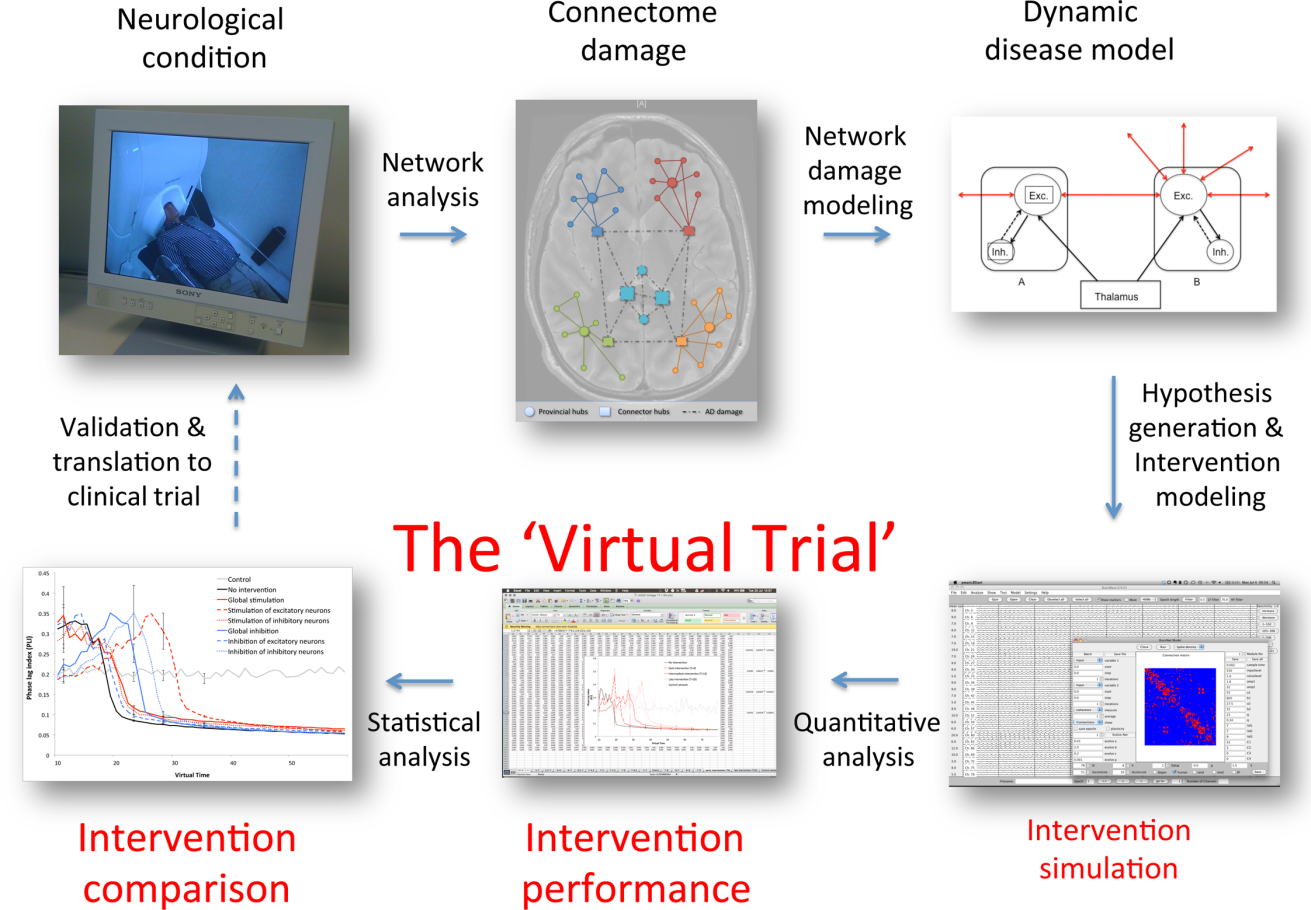
Global overview of relevant modeling and analysis procedures. This study focuses on the parts indicated in red: the virtual trial. The general workflow of our analysis can be described as follows: the dynamic network model is run with the degeneration algorithm and, simultaneously, one of the interventions (or no intervention). Hence, the network is damaged over time according to local neuronal activity levels, but at the same time, by changing neuronal excitability levels due to varied threshold potential (Vd) settings (see below for details), a counterstrategy is employed to diminish the effect of the damage and maintain network topology close to the original state. The resulting oscillatory, connectivity and network topology changes are then described using the selected measures (see below) to evaluate the effect of the different interventions over time, and finally these are compared statistically to obtain an impression of the most successful strategy. For a more detailed stepwise description of the analysis, please refer to the Method section.

Despite the theoretical advances, complex network analysis of the brain has not yet led to any improvement for AD patients. Although abstract by nature, in our opinion network modeling might facilitate a transition towards clinical applications: in addition to simulating degenerative or lesion effects, models may also be employed to explore therapeutic intervention strategies, in order to make dynamic network changes more predictable and easier to understand in terms of their underlying mechanisms. Therefore, we used our computational AD degeneration model for the present study, but now with the addition of various ‘therapeutic’ network interventions. In the model, neuronal excitability levels of both excitatory and inhibitory neurons can be adjusted selectively (roughly simulating medication effects or nonpharmacological stimulation/inhibition techniques). Since neuronal hyperactivity was the main driver of network degeneration in this model, we hypothesized that the strategy employing global neuronal inhibition would be most effective in countering this process. Fig 1 provides an overview of the overall workflow.

## Results

**Fig 2:**
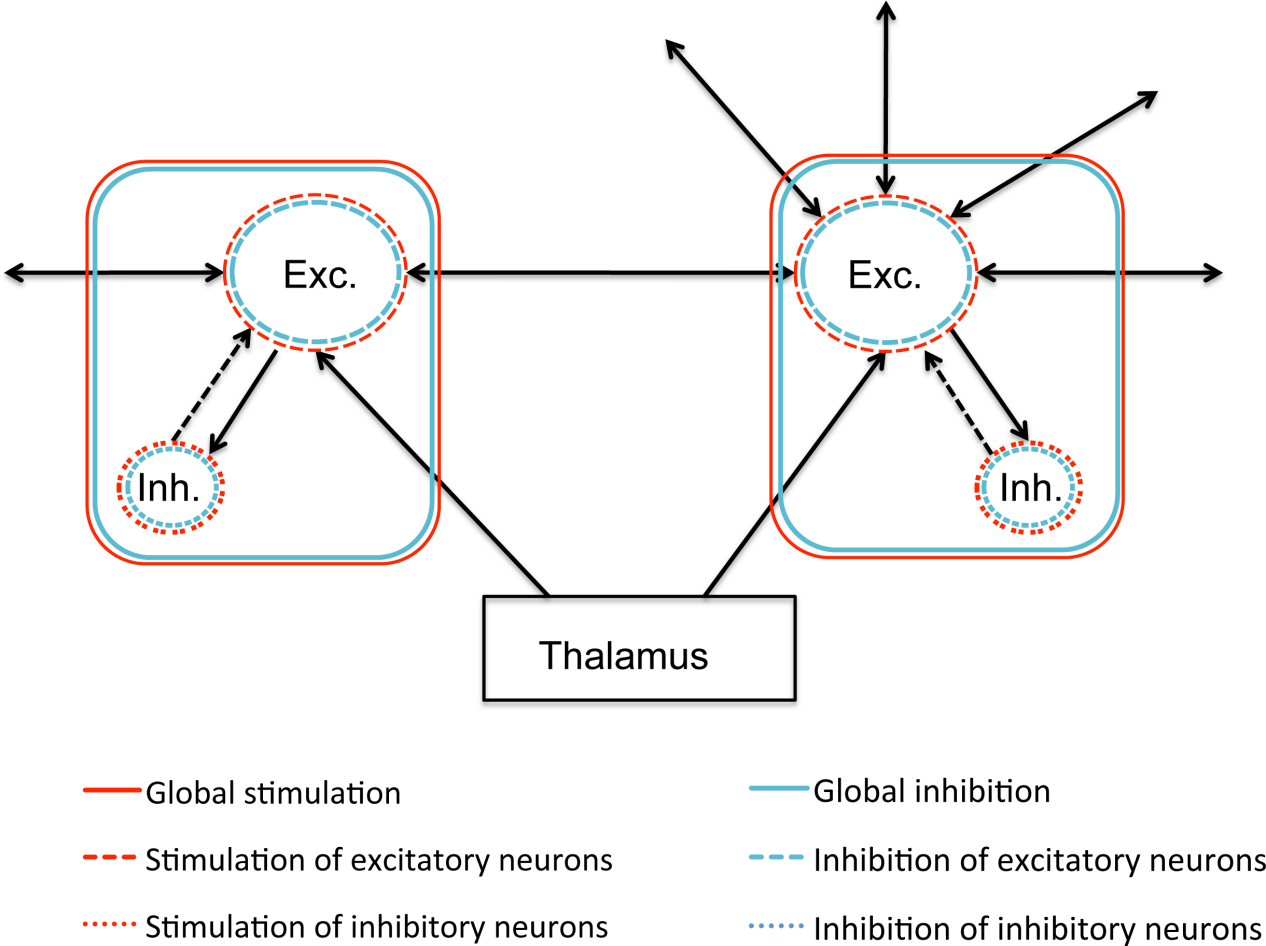
Influence of intervention strategies on the neural mass model. All black lines are connections that are damaged by the ADD mechanism. Note that due to the interplay between excitatory and inhibitory forces and the network topology, the effect of an intervention on a network level is not easy to foresee. All strategies were continuous. Exc. = excitatory neurons, Inh. = inhibitory neurons.

In each experiment, six intervention strategies were compared to the ‘no intervention’ (degeneration on, no intervention) and ‘control’ (no degeneration, no intervention) conditions: global stimulation, global inhibition, selective stimulation or inhibition of all excitatory neurons, and selective excitation or inhibition of all inhibitory neurons (see Fig 2). Different levels of stimulation and inhibition were tested (see method section). Within the individual strategies, adjusting the excitability levels produced fairly similar results (i.e. different threshold potential (Vd, see method section and S2 Text) and for clarity purposes we chose a single representative value for each strategy to be depicted in the figures below.

To assess functional network status during the degeneration period, several relevant quantitative markers were employed, highlighting three main aspects of functional network quality: spectral power, functional connectivity and network topology (see Table 1). In addition, the effect of degeneration on structural connectivity was compared between the most successful strategy and the ‘no intervention’ mode, using the normalized node strength (see method section). Using this strategy, the effect of intervention timing was also explored. Note that all intervention strategies were commenced after ten degeneration cycles (T = 10), to simulate a disease condition for which therapy was started with a delay (see also figure 6 of [18]), except in the intervention timing tests (experiment 6). Graphs depict *only* the intervention period.

**Table 1:** Overview of network analysis measures. Measure selection was based on the aim to describe the most robust and relevant functional network changes in AD. For exact definitions please refer to the method section and Supporting Information S4.

Category	Measure	Interpretation
**Oscillatory behavior**	Relative alpha power	Average relative amount of spectral power in the lower alpha frequency band (8–10 Hz)
	Peak frequency	Dominant frequency in the power spectrum, averaged over all nodes in the network
**Functional connectivity**	Phase Lag Index (PLI)	Average functional connectivity strength of all individual nodes in the network.
**Network topology**	Local connectivity: Clustering coefficient	Average, normalized, weighted functional connectivity level between individual network nodes and their environment (directly connected nodes); an indicator of local network connectivity strength.
	Community detection: Modularity index	Indicator of community (module) presence in the network. A high value signifies a network easily dividable in subnetworks.
	Robustness: Algebraic connectivity (Graph spectral analysis)	Overall connectivity strength of the network, based on graph spectral analysis. Indicator of network robustness and resilience against damage.
	Hub presence: Leaf number (Minimal spanning tree analysis)	Measure of overall functional network structure, based on minimum spanning tree analysis. A network with a higher leaf number has a more star-like architecture, with high degree hubs.
**Structural connectivity**	Normalized node strength	Level of structural connectivity (averaged over all edges) of individual nodes, divided by their original level. Indicates the change in structural network connectivity over time.

### Experiment 1a: Spectral analysis–relative alpha power

**Fig 3:**
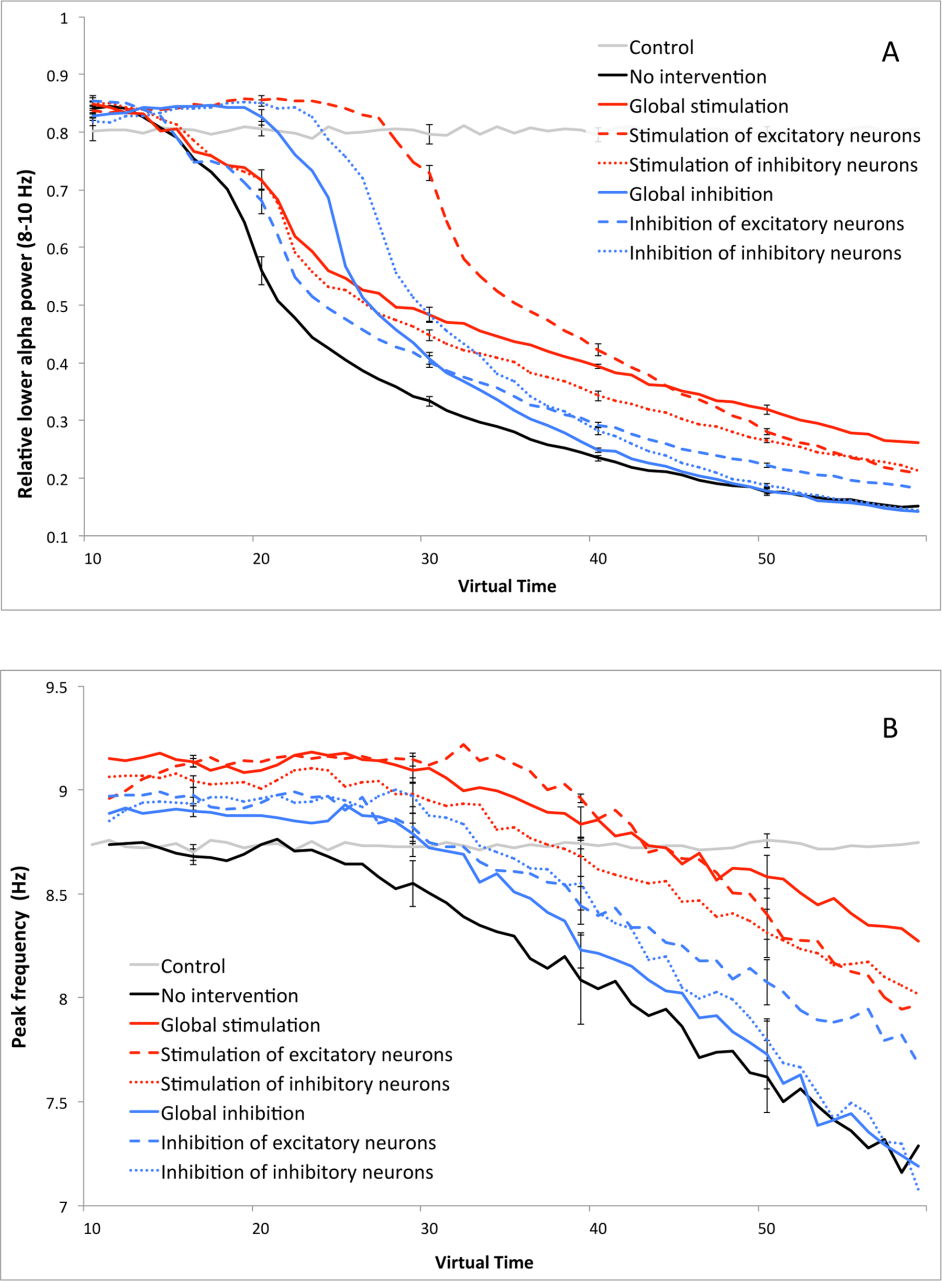
Spectral analysis. A) Comparison of relative lower alpha (8–10 Hz) power values over time for the various interventions. B) Comparison of peak frequency level over time for the various interventions. Error bars indicate the standard deviation over the total number of runs of the various strategies.

Relative power in the lower alpha band (8–10 Hz) inevitably decreased during the degeneration process, but the rate at which this happens differed substantially between the various strategies (see Fig 3A). Without intervention (black line), lower alpha power breaks down around T = 20, decreasing to a level between 0.1 and 0.2. Global inhibition, inhibition of inhibitory neurons, and especially stimulation of excitatory neurons was able to prolong a normal level of relative power in the lower alpha band (around 0.8) and postpone the subsequent collapse.

### Experiment 1b: Spectral analysis–peak frequency

The gradual decrease of the (posterior) alpha peak value is a robust finding in AD, and levels lower than 8 Hz are generally regarded as pathologic [48]. Without intervention, the alpha peak is stable for a short while, but starts to decrease steadily around T = 25. The same pattern was observed during all intervention strategies, although the peak level stayed above 8 Hz for a substantially longer period. This seems to be mainly due to a higher initial peak value (Fig 3B).

### Experiment 2: Functional connectivity–Phase Lag Index (PLI)

**Fig 4:**
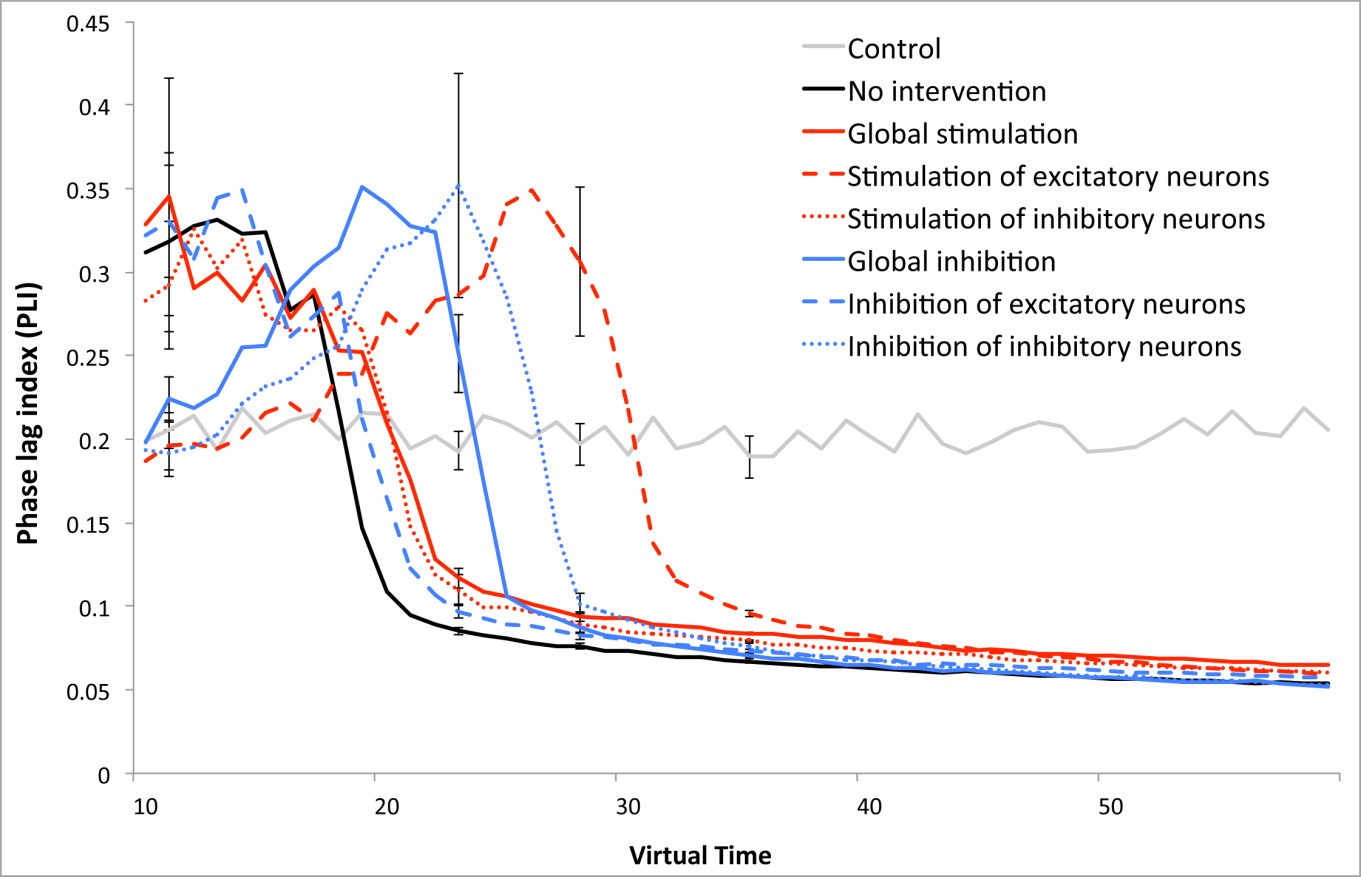
Functional connectivity analysis: comparison of Phase lag index (PLI) values over time for the different interventions. Error bars indicate the standard deviation over the total number of runs of the various strategies.

Overall functional connectivity in the network showed a critical transition period around T = 20 with a breakdown even more abrupt than in the lower alpha power results (see Fig 4). In the ‘no intervention’ condition, PLI values were much higher than in the control network at the start of therapy due to the ADD process, but it rapidly fell to low levels. The ‘stimulation of excitatory neurons’ regime was able to maintain normal/high PLI values most successfully, but suddenly dropped down to the same level as the other scenarios around T = 30.

### Experiment 3a: Network topology–Local connectivity

**Fig 5:**
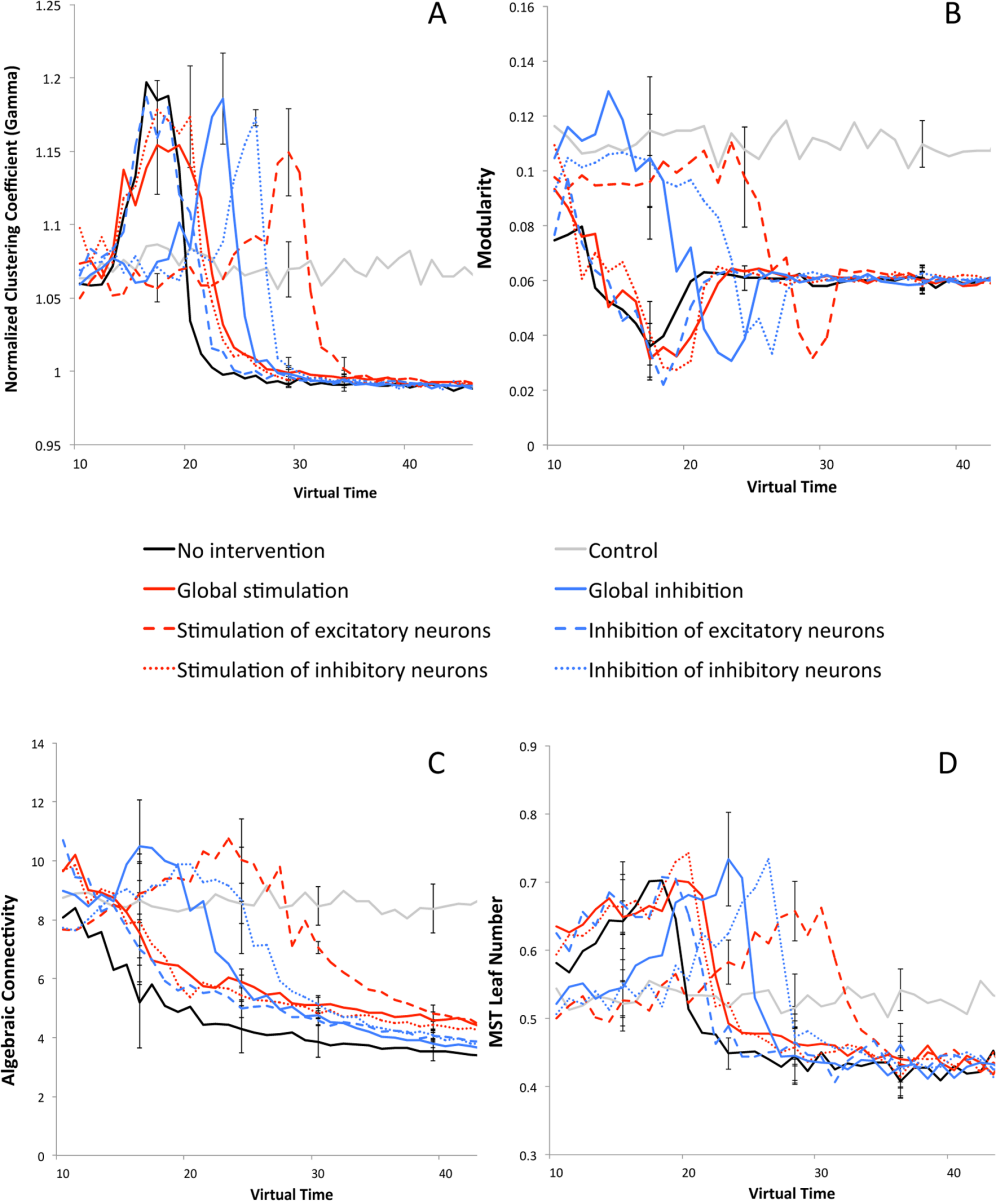
Network topology. A) Network analysis: comparison of normalized clustering coefficient (gamma) values over time for the different interventions, indicating local connectivity strength. B) Comparison of modularity (community presence) over time for the different interventions. C) Comparison of algebraic connectivity values over time for the different interventions, indicating network robustness. D) Comparison of MST leaf number values over time for the different interventions, reflecting network hub presence. Error bars indicate the standard deviation over the total number of runs of the various strategies.

AD network degradation as assessed by EEG/MEG has previously been characterized by a decrease of the (normalized) clustering coefficient [49,50], contributing to the loss of presumably efficient small-world network topology. Here, a pattern similar to the PLI results developed, and with the global inhibition, inhibition of inhibitory neurons and stimulation of excitatory neurons as most beneficial regimes (Fig 5A).

### Experiment 3b: Network topology–Modularity

Macroscopic brain connectivity networks have been shown to possess a (hierarchical) modular structure, a feature that is presumed to promote network efficiency and robustness. In AD, modularity gradually weakens [51]. ‘Stimulation of excitatory neurons’ seemed to retain normal modular structure relatively well (Fig 5B).

### Experiment 3c: Network topology–Robustness

Algebraic Connectivity is a graph spectral measure of overall network robustness, higher values signifying a network that is harder to tear apart [52,53]. Strategies that were relatively successful in upholding the robustness of the network were ‘global inhibition’, ‘inhibition of inhibitory neurons’ and ‘stimulation of excitatory neurons’ (Fig 5C).

### Experiment 3d: Network topology–Hub presence

It has been reported previously that network hub structure is lost in AD [54]. In this simulation similar findings became apparent, with a later decrease of the MST Leaf number in the most successful scenarios, ‘inhibition of excitatory neurons’, ‘global inhibition’ and ‘stimulation of excitatory neurons’.

### Experiment 4: Structural network connectivity–Normalized Node Strength

**Fig 6:**
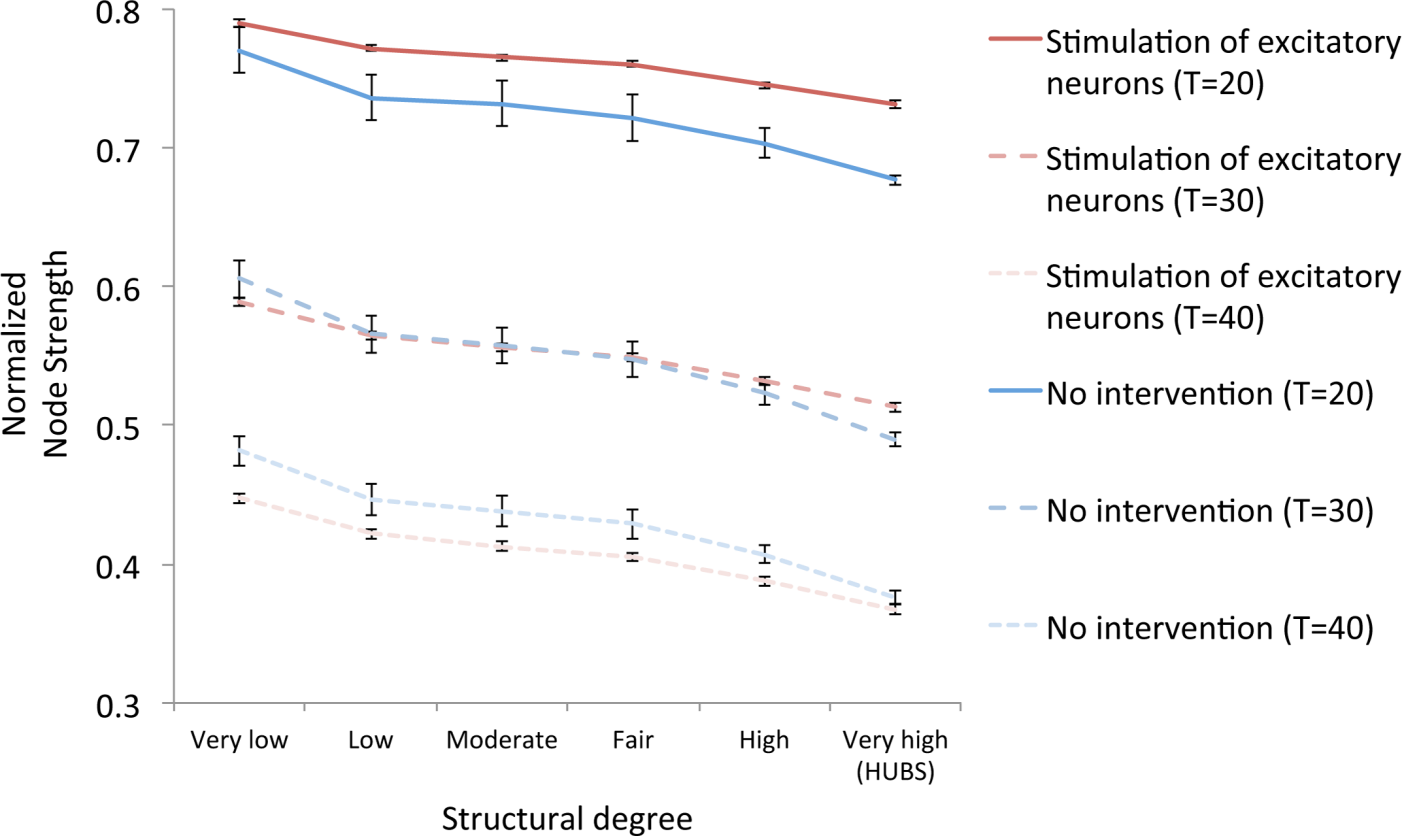
Effect of the ‘stimulation of excitatory neurons’ intervention on structural connectivity. At three different time points, structural connectivity strength is compared with the ‘no intervention’ condition (blue lines). This is done in six different categories, based on structural connectivity level (degree). Highly connected hubs fall in the sixth category, most right in the chart. Note: error bars indicate standard deviations based on all node degrees per category. For a detailed list of region names and degree distributions see [18, Table 1].

Structural connectivity decreases inevitably over time in the model, but this process can be delayed by interventions. Fig 6 compares the ‘no intervention’ condition (blue lines) with the relatively successful stimulation of excitatory neurons (red lines). For different time points normalized node strength is plotted against original structural degree. This measure is the ratio of present structural degree over its original degree, so a value smaller than 1 indicates a loss of structural connectivity. The loss was not equal for all nodes; hubs tend to decrease more, confirming the previously reported hub vulnerability (18,49,55). The result of the intervention was that initially damage is counteracted better (see T = 20), but ultimately the network apparently suffered more than in the control state (see T = 40). In the intervention condition hub vulnerability is less pronounced; the slope of the lines is less steep. Remarkably, at T = 30, overall structural connectivity was fairly equal between the conditions, while functional measures differed considerably (see results above).

### Experiment 5: Intervention timing

**Fig 7:**
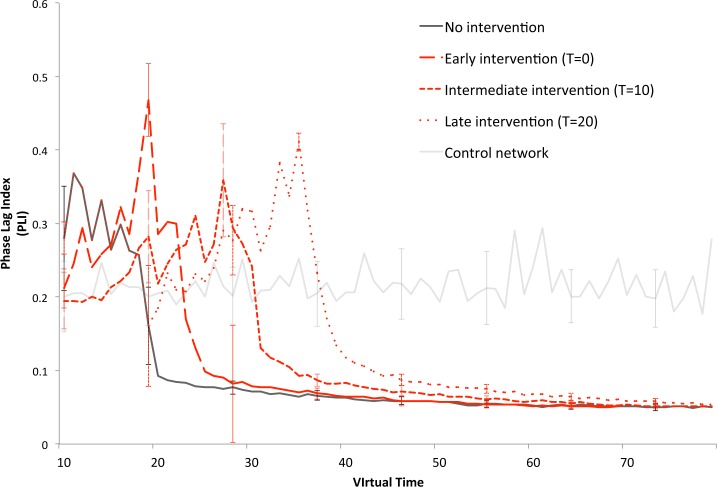
Intervention timing. Overall functional connectivity (Phase Lag Index, PLI) over time is compared between the ‘no intervention’ condition, a healthy control network, and the most successful intervention, ‘stimulation of excitatory neurons’, started at three different points in time. Note that the graph starts at T = 10; the ‘early’ intervention has been active ten cycles before this graph starts. Error bars indicate the standard deviation over the total number of runs of the various strategies.

To assess the effect of the specific delay with which the intervention is started, we compared functional connectivity results (PLI) for three different intervention starting points (T = 0, T = 10 and T = 20), using the relatively successful ‘stimulation of excitatory neurons’ strategy. As can be judged from Fig 7, the response of the PLI to the differently timed interventions was similar, and their ability to uphold PLI values near or above the control network levels seems mainly dependent on the moment in the degeneration process at which the intervention was initiated.

### Overall intervention performance

**Fig 8:**
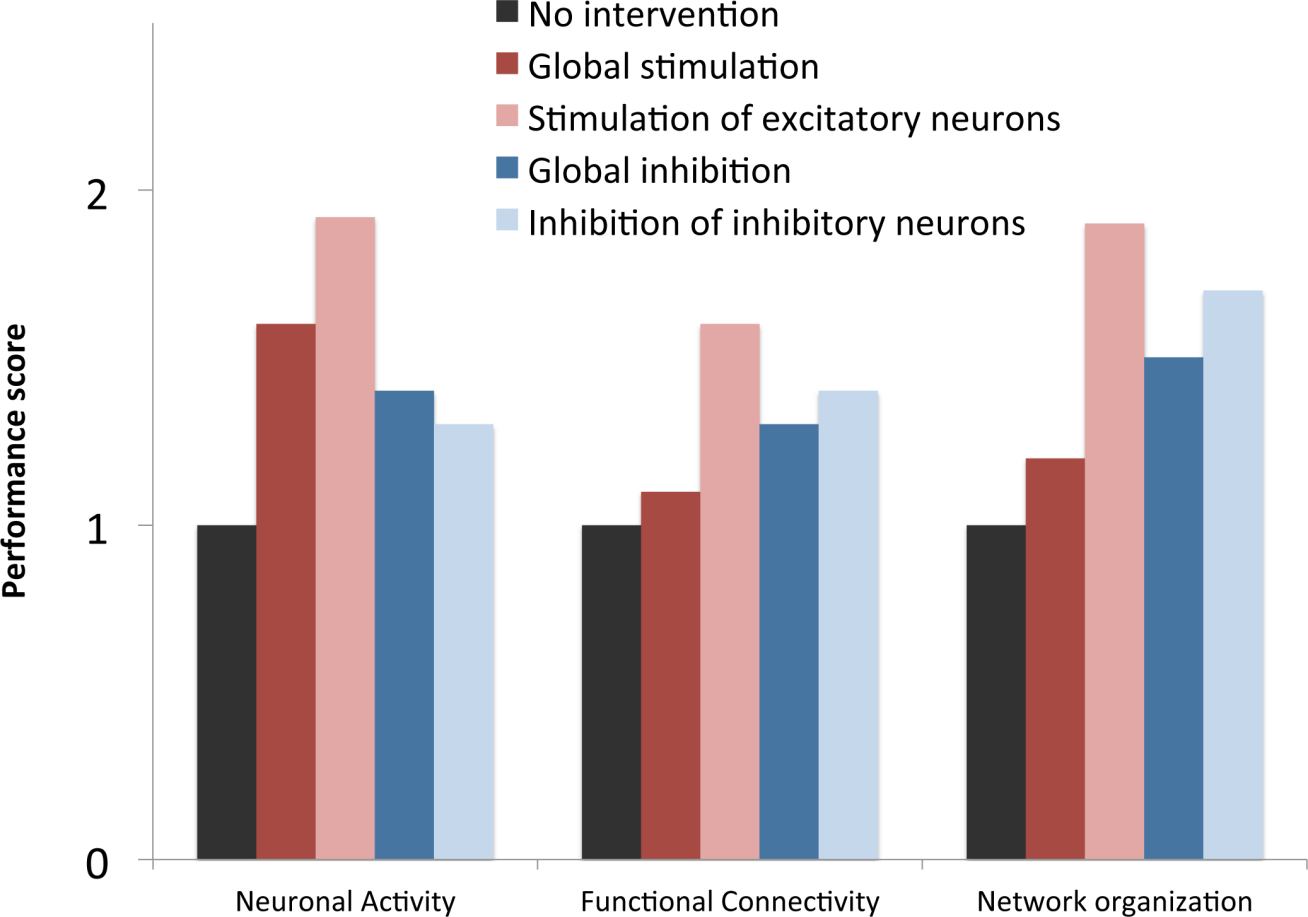
Overall intervention performance. Performance score of the 4 most successful strategies compared to the ‘no intervention’ condition. Bar height is based on a total score per category, derived by summing the amount of time that measures within that category stay near a normal level, which is then divided by the ‘no intervention’ score of that category to derive a performance ratio.

Overall performance of the four most successful strategies was assessed by combining results into a total sum per category for the four most successful interventions (Fig 8, see caption or method section for score definition). This enables a comparison at a glance between the various strategies regarding different qualities, and show that all these strategies bring an overall improvement compared to the ‘no intervention’ state, with individual differences. The most successful strategy was the ‘stimulation of excitatory neurons’ intervention, but also inhibitory strategies performed fairly well.

## Discussion

In this study we used a simplified neurophysiological human brain network model to investigate the effects of altering neuronal excitability during an activity-dependent degeneration scenario that produces AD-like functional network deterioration. We conclude that, in this computational model, the most successful strategy to preserve network integrity is, contrary to our hypothesis, a selective stimulation of excitatory neurons. A virtual network intervention approach as described here may help to predict the impact of interventions on the human brain.

### Explaining the success of neuronal stimulation in a hyperactive network

The observation that the ‘stimulation of excitatory neurons’ strategy is most successful in retaining network organization in a situation where neuronal activity leads to damage seems contradictory at least, and is in stark contrast with our initial hypothesis. Intuitively, one would be inclined to choose a strategy to slow down the degeneration process by either inhibiting excitation or by stimulating inhibition, and, judging from our results, the inhibitory strategies perform quite well. Several pharmacological studies aimed at countering aberrant neuronal hyperactivity provide circumstantial support for this view [31,55]. However, as can be judged from the present experiments, the effects of the different types of stimulation and inhibition are quite unpredictable: in this particular topology, the increased excitability in excitatory neurons apparently leads to a condition where desirable network topology is retained longer. Although this result begs for an mechanistic explanation, this was not the primary goal of our current experiment. However, we did perform further analyses to better understand the outcome (see also S3 Text): we hypothesized that one reason for a beneficial effect might be that the net effect of stimulation of excitatory neurons on the entire neural mass is inhibitory, due to the interplay with the relatively influential inhibitory neuron groups. However, analysis showed that this is not the case. Since all neural masses are equal except for their connectivity pattern, the explanation may lie in the network topology itself. For example, the successful scenarios seem to suppress functional hub strength of the network (see Fig 5D), and while hubs may generally improve network efficiency, they can also facilitate the spread of pathology[56]. Different topologies should be tested with the same degeneration algorithm in future experiments to explore this idea. An alternative explanation, is that the philosophy behind a strategy should perhaps not be based on trying to oppose the degeneration mechanism, but on keeping the network ‘alive’ for as long as possible in general by stimulating the remaining neurons in a decaying network; compensatory activation. However, inhibitory strategies performed quite well in our experiments, and current literature clearly points to the pathological aspect of the hyperactivity in AD [57–59].

### Strategy selection and timing

With the present degeneration algorithm, intervention success is relative, since regardless of the chosen strategy, network breakdown was inevitable. This may be explained by specific features of the algorithm, as described in the study limitations paragraph. Another issue is our selection of strategies. As a first exploration we opted for global, continuous interventions, but many more scenarios are possible, and might lead to different outcomes: selective stimulation or inhibition of specific regions (for example hub regions), different delay or timing of interventions, interventions based on individual connectivity patterns, adaptive interventions, structural interventions, and so on. A present-day major objective in the field of neurodegenerative disease research is the aim for early intervention: there is a growing consensus that, to be successful, therapy should be commenced before structural pathology is widespread [60]. We examined the influence of three different intervention starting points (see Fig 7) on functional connectivity (PLI), an outcome measure that is the basis of all graph theoretical analysis, and a measure that produced clear differences among the various strategies. Here however, no clear benefit of early intervention initiation is found; in fact, the response of the network to the ‘stimulation of excitatory neurons’ intervention seems independent of the moment in de degeneration process. This is surprising, since underlying structural connectivity and topology is inevitably weaker at later time points, raising the question whether some kind of adaptive therapy could be effective, i.e. starting stimulation at low PLI levels, and stopping at high levels.

Another intriguing finding was the sharp peak in activity and connectivity just prior to sudden decrease in value in several of the scenarios, including the most successful ones, reminiscent of a non-linear system near a critical transition (close to breakdown in this case). Although we did not elaborate on this, this finding may also point to the potential merit of an adaptive strategy that can avoid the increases and thereby prevent network collapse.

### The definition of a successful strategy

Also, the success of a therapy depends on how ‘success’ is defined. In this exploratory study, we adhered to the general idea of keeping a system in or close to its original functional state over time, which seems straightforward and in line with goals in medical practice in general. Of course, in our model, the virtual time base does not permit statements about actual timelines or tempo of deterioration, but when a strategy is able to uphold certain desired characteristics approximately twice as long (see for example Fig 4) as in the ‘no-intervention’ state, this can reasonably be thought of as successful; real-life therapy that delays degenerative damage effects in AD this long would be considered a spectacular improvement. Although a translation of our present findings to clinical experiments and pharmacological recommendations is too ambitious at this point, an appealing thought is that the current medication for AD leads to stimulation of excitatory neurons, the most successful strategy in our experiments as well. Ultimately, intervention modeling studies will require validation through real interventions targeting brain activity, to evaluate their true value.

### Study limitations

Various arbitrary choices that were made in this exploratory study may have had an influence on its outcome. For example, one might wonder whether the model is sufficiently detailed to realistically simulate cerebral dynamics. Neural masses were identical (except for their connectivity characteristics), while there are distinct regional differences in the brain. Also, the number of masses (n = 78) could be augmented based on more recent and detailed structural connectome datasets. Our present DTI-based structural connectivity data in the model is based on deterministic tractography, which has been demonstrated to be inadequate for detecting crossing white matter fibers, and newer, more powerful methods are available [61,62]. Repeating the current approach using alternative connectome data is an important way to validate model findings, and should be performed in future studies. Another interesting recent approach is to replace the structural network by one that is based on a generative model itself [63–65]. Also, one could argue that connectome data taken from an older control group would have led to different, more representative results. Furthermore, the neural masses themselves model a neuronal region macroscopically, but not on a microscopic level, while detailed neurophysiological models at (sub)cellular level are certainly available. Could more detail have led to a different outcome? While that certainly is possible, we have several motives for our current approach. First, the global macroscopic level of analysis is of particular interest, since clinical measurements (e.g. EEG, (f)MRI) are at this level; detailed in vivo measurements on a microscopical level in memory clinic patients are not (yet) feasible. Second, the observation that, even in this relatively basic model of human connectivity, the outcomes of fairly straightforward interventions defy an easy mechanistic explanation underscore the complexity of network effects, and the need for modeling. Third, data analysis time and computing power are finite, and added detail can increase demands placed on these aspects exponentially.

Other limitations stem from specific choices that are made in the activity-dependent degeneration (ADD) algorithm that was used in this study. The algorithm was chosen because it is based on the singular assumption that neuronal hyperactivity leads to dysfunction and damage, and because of its strong resemblance with Alzheimer-related network degeneration, including features like early-stage disinhibition and hub vulnerability [18]. However, in the ADD algorithm the network is damaged regardless of the absolute level of activity; even regions with a normal level of activity will be damaged, although exponentially less (see ‘loss function’ in S1 Text). A degeneration regime that spares regions that exhibit a normal range of neuronal activity might be more plausible (metabolically), and could result in strategies that will be able to maintain network integrity for a longer, perhaps even indefinite, period of time. Also, neuronal plasticity as a defense mechanism to degeneration was not incorporated in this model, but may yield more realistic results and enhance network survival. For example, mechanisms such as synchronization-dependent or growth-dependent plasticity could be implemented in de model [66]. Our rather ‘unforgiving’ degeneration mechanism may have underestimated the impact of the various therapies, and alternative algorithm choices may lead to different intervention outcomes. However, adding more assumptions (and complexity) to the model or its degeneration algorithm will probably enlarge the unpredictability of the interventions. Finally, the chosen Vd stepsize of 0.5 in our analysis was a pragmatic choice, and although our Vd analysis in the Supporting Information (S2 Text) suggests that Vd values in between would not produce very different results, a smaller stepsize may have been more insightful.

### Clinical relevance and future directions

With the growing interest in the human connectome in general, and more specifically in connectivity as a biomarker of neurodegenerative disease [67,68], intervention modeling is a logical step forward. Computer simulations serving as a virtual test ground for intervening in complex systems is common practice in related fields, such as economy, meteorology or systems biology. With the advent of enhanced acquisition techniques such as DTI and MEG, enabling increasingly accurate human connectivity datasets, and of increasing computational power, analysis will become more elaborate, faster and hopefully more user-friendly and available to clinical researchers. Two long-term aims seem meaningful to pursue: the further development of dynamic connectome (“dynome”) intervention models, and the validation of these models with clinical experiments, perhaps involving individual structural connectome data to achieve personalized results. This way, the modeling approach may become a viable intermediate step in the bidirectional interpretation of both etiological hypotheses and clinical experiments in connectivity-related research in general [56].

### Conclusion

The observations in this computational modeling study surprisingly suggest that AD-like network degeneration due to neuronal hyperactivity can be countered most effectively by global stimulation of excitatory neurons. The wide-ranging and unpredictable impact of intervention strategies in this straightforward dynamic human connectome model, with a limited number of identical neural masses and macro scale cerebral topology, illustrates the fact that interventions in complex systems can lead to counterintuitive results. In general, network intervention analysis may help to explore and explain therapeutic effects aimed at preserving network integrity, and thereby potentially guide the design and hypothesis selection of clinical intervention trials.

## Methods

### Study design

For this study we simulated neurophysiologic activity of cortical regions embedded in a realistic structural network topology to evaluate hypotheses about the relation between neuronal activity and (structural and functional) connectivity. The output of this model provides information about neuronal activity in the form of average voltage and spike density per region, and generates EEG-like data that can be subjected to further analysis. Furthermore, hypotheses about brain pathophysiology can be tested by artificially changing structural or dynamical properties of the model. The general workflow of our analysis can be described as follows (see also Fig 1 for a graphical overview): the dynamic network model is run with the degeneration algorithm and, simultaneously, one of the interventions (or no intervention). This means that the network is damaged according to local neuronal activity levels, but at the same time, by changing neuronal excitability levels due to varied threshold potential (Vd) settings (see below for details), a counterstrategy is employed to diminish the effect of the damage and maintain network topology close to the original state. The resulting oscillatory, connectivity and network topology changes are then described using the selected quantitative outcome measures (see below) to evaluate the effect of the different interventions over time, and in the final step these are compared statistically to obtain an impression of the most successful strategy.

### Network dynamics: Description of the Neural Mass Model

We used a model of interconnected neural masses, where each neural mass represents a large population of excitatory and inhibitory neurons generating an EEG (or MEG) like signal. The model was recently employed in two other graph theoretical studies [18,69]. The basic unit of the model is a neural mass (NM) of the alpha rhythm [70–72]. This model considers the average activity in relatively large groups of interacting excitatory and inhibitory neurons. Spatial effects (i.e. distance) are ignored in this model; brain topology is introduced by coupling multiple NMs together. The average membrane potential and spike density of the excitatory neurons of each of the NMs separately were the multichannel output that was subject to further analysis.

### Network structure: Human network topology

A diffusion tensor imaging (DTI) based study by Gong et al. published in 2009 that focused on large-scale structural connectivity of the human cortex resulted in a connectivity matrix of 78 cortical regions [73,74]. The connectivity matrix was implemented in our model software, and used as topological framework for the 78 coupled NMs. Coupling between two NMs, if present, was always reciprocal, and excitatory. Note that at the start of the simulation, the coupling strength between all NM pairs was identical, and the only difference between the cortical regions (or NMs) was their degree of connectivity to other NMs (cortical regions). Please refer to the supporting information for full details.

### Network degeneration: Activity-dependent degeneration (ADD)

The neural mass model described above was extended to be able to deal with activity dependent evolution of connection strength between multiple coupled NMs. Activity dependent degeneration (ADD) was realized by lowering the ‘synaptic’ coupling strength as a function of the spike density of the main excitatory neurons (all neural mass model parameters and functions are summarized and explained in S1 Text), in a similar way as previously described (18). The effects of ADD were measured by changes in ‘total power’ (local average membrane potential) and spike density, and these two measures were used as representations of neuronal activity in further analyses. The computational model is incorporated in our custom developed analysis software (‘BrainWave’, v0.9.151.5), written by C.J. Stam (available for download at http://home.kpn.nl/stam7883/brainwave.html).

### Network defense: Adjustment of neuronal excitability thresholds

For the present study, we introduced counterstrategies against ADD that involve altering the neuronal excitability, either at a global level or of excitatory or inhibitory neural masses selectively. In the neural mass model, a transfer function determines the translation of membrane potential to spike density and vice versa (see S1 Text). Vm is the average membrane potential, and Vd is the threshold potential (Vd1 for excitatory, Vd2 for inhibitory neurons). Altering the level of Vd results in a sigmoid function describing the resulting spike density (see S1C Fig); a lower threshold leads to a higher spike density and vice versa. This way, the excitability of a neural mass can be changed either for excitatory or inhibitory groups selectively, or for both simultaneously.

In this model, extremely low or high neuronal excitability levels (Vd1 or Vd2 lower than 4 or higher than 10,) cause the system to quickly reach non-functional states, either shutting down functional connectivity or generating cascades of uncontrolled activity, respectively (reminiscent of epileptic seizure activity). Therefore, these were excluded from further analysis. Within the biologically plausible range, we tested various excitability levels and their effect on network dynamics (see also Fig 2). For clarity purposes, we limited the number of strategies to six distinct types: global (both excitatory and inhibitory) stimulation (Vd1 = 6, Vd2 = 6), global inhibition (Vd1 = 8, Vd2 = 8), selective stimulation of excitatory neurons (Vd1 = 5, Vd2 = 7), selective stimulation of inhibitory neurons (Vd1 = 7, Vd2 = 5), selective inhibition of excitatory neurons (Vd1 = 8, Vd2 = 7), and selective inhibition of inhibitory neurons (Vd1 = 7, Vd2 = 6.5). The strategies consisted of maintaining constant neuronal excitability levels; the initial settings of a strategy did not change over time during the degeneration period. Since for each of these interventions the threshold potential adjustment is arbitrary, we conducted simulations with six different values within each category (for example, for global stimulation six Vd1 settings between 4 and 6.5 were used, with a 0.5 increment), and compared the findings to pick a representative value. See S2 Text for an illustration of this analysis. All intervention strategies were initiated after ten degeneration cycles without any therapy, at T = 11, to simulate the manifestation of disease and subsequent therapy initiation with some delay. Model output is generated for >50 cycles of the degeneration algorithm, to simulate a progressive neurodegenerative process over time. Therefore, the X-axis variable has been defined as ‘virtual time’. Note that there is no defined relation to real time; this parameter should not be interpreted as hours, days or otherwise. In this study, we compared relative differences within the model.

### Outcome measures

#### Spectral analysis

As spectral analysis is a common quantitative neurophysiological procedure that provides clinically relevant information in neurodegenerative dementia, we included this in our experiments. Fast Fourier transformation of the EEG-like oscillatory output signal was used to calculate relative power. Since this neural mass model mainly generates oscillatory activity in the alpha range, the relative power analysis results should not be directly compared to patient data, but mainly serve to illustrate the changes over time in the model. Relative lower alpha power and peak frequency were included because of the well-known gradual decreases observed in AD patients[1].

#### Functional connectivity analysis

The Phase Lag Index (PLI) is a measure of the asymmetry of the distribution of phase differences between two signals. It reflects the consistency with which one signal is phase leading or lagging with respect to another signal [41]. The PLI performs at least as well as the synchronization likelihood (SL) in detecting true changes in synchronization but it is much less affected by the influence of common sources [75].

The PLI is defined as index of the asymmetry of the phase difference distribution by means of

PLI=|〈sign[sinΔϕ(t)]〉|

in which *t* refers to time. If the phase difference Δ*ϕ* between two signals is in the interval 0 < Δ*ϕ* < *π*, the sinus function will produce a positive, non-zero value. The PLI is bounded 0 ≤ PLI ≤ 1 and a PLI of zero indicates either no coupling or coupling with a phase difference centred around 0 mod *π*, which may be caused by volume conduction. On the other hand, the stronger the non-zero phase locking is, the larger the PLI will be; a PLI of 1 indicates perfect phase locking, i.e. functional connectivity. For this study, PLI is calculated for all pairwise nodes and then averaged, resulting in one global value per analysis.

#### Graph theoretical analysis

Network topology measures were included in our analysis based on previous graph theoretical literature in AD, and on previous experience. Although measure definition and network comparison is a methodological challenge, our choice of measures aims to give an overall impression of changing network topology. To characterize relevant changes in functional network organization we examined four aspects: the connectivity strength between neighboring nodes (normalized weighted clustering coefficient or ‘gamma’ [49]), the existence of subnetworks (modularity [66,76], the robustness against damage (algebraic connectivity [52,53]), and the existence of hub structure in the network (minimum spanning tree leaf number analysis [77]). To investigate the effect on structural network topology, we looked at the decrease in structural connectivity in nodes compared to their original connectivity strength (normalized node strength [18]). All used measures are summarized in Table 1.

#### Statistical analysis

The various intervention strategies were compared with a ‘no intervention’ condition, in which the ADD algorithm was used without countering intervention, and with a ‘control’ condition, in which the simulation was run without ADD algorithm or intervention. In all conditions the structural and dynamic network at baseline was completely identical, and the only changes made were the inclusion of the ADD degeneration algorithm and the adjustment of the membrane threshold potential (Vd) value corresponding to the specific strategy.

To enable statistical comparison between the various intervention strategies with the ‘healthy control’ situation and with the ADD simulation without intervention, all different conditions were simulated ten times. These results were averaged to obtain a single representative value for each measure (for each time point). For the measures that produce results per node, the overall mean value of the whole network was used for further analysis. Standard two-tailed t-tests were used to obtain significant differences between strategies and the control conditions. A measure was considered within a ‘normal’ range when it was not significantly lower than the control condition at a certain point in time.

For evaluation and comparison of the strategies at a glance, we have summarized their results per category (neuronal activity, functional connectivity, network topology) in a global ‘performance score’ (see Fig 7). This score is derived by summing the amount of virtual time that markers within that category stay above or near a normal level (i.e. are not significantly lower than the ‘control’ condition), which is then divided by the ‘no intervention’ score of that category to derive a performance ratio.

## Supporting information

S1 Fig (A) Schematic presentation of single neural mass model. The upper rectangle represents a mass of excitatory neurons, the lower rectangle a mass of inhibitory neurons. The state of each mass is modeled by an average membrane potential [*V*_e_(*t*) and *V*_i_(*t*)] and a pulse density [*E*(*t*) and *I*(*t*)]. Membrane potentials are converted to pulse densities by sigmoid functions *S*_1_[*x*] and *S*_2_[*x*]. Pulse densities are converted to membrane potentials by impulse responses *h*_e_(*t*) and *h*_i_(*t*). *C*_1_ and *C*_2_ are coupling strengths between the two populations. *P*(*t*) and *Ej*(*t*) are pulse densities coming from thalamic sources or other cortical areas respectively. (B) Coupling of two neural masses. Two masses are coupled via excitatory connections. These are characterized by a fixed delay *T* and a strength *g*. (C) Essential functions of the model. The upper left panel shows the excitatory [*h*_e_(*t*)] and inhibitory [*h*_i_(*t*)] impulse responses of Eq. 1. The upper right shows the sigmoid function relating average membrane potential to spike density (Eq. 2). (D) Overview of neural mass model parameters.
(DOC)

S2 Fig (A) The effect of Vd level on lower alpha relative power during the ‘stimulation of excitatory neurons’ scenario. (B) The effect of Vd level on peak frequency during the Stimulation of excitatory neurons scenario. (C) The effect of varying Vd levels on PLI during the 'Stimulation of excitatory neurons' strategy. A lower Vd1 seems related to a longer lasting normal PLI level. (D) The effect of Vd level on the normalized clustering coefficient (gamma, a measure of local connectivity) during the 'Stimulation of exctiatory neurons' scenario. (E) The effect of Vd level on algebraic connectivity (robustness) during the 'Stimulation of excitatory neurons' scenario. (F) The effect of Vd level on modularity (subnetwork presence) during the 'Stimulation of excitatory neurons' scenario. (G) The effect of Vd level on MST Leaf Number (network hub presence) during the 'Stimulation of excitatory neurons' scenario.
(DOC)

S3 Fig Comparison of total power values (as a measure of neuronal activity) over time during the "Stimulation of excitatory neurons' scenario (blue) and the 'No intervention' state (red). (TIF)

S1 Text The Neural mass model. (DOC)

S2 Text Membrane threshold potential (Vd) selection. (DOC)

S3 Text Single neural mass activity comparison between the ‘Stimulation of excitatory neurons’ scenario and the ‘No intervention’ state. (DOC)

S4 Text Graph measure definition. (DOCX)
