# Supplementary figures and images for: Altering neuronal excitability to preserve network connectivity in a computational model of Alzheimer's disease
Source: PLoS Comput Biol. 2017 Sep 22;13(9):e1005707. doi: 10.1371/journal.pcbi.1005707 (PMC5627940; doi:10.1371/journal.pcbi.1005707)

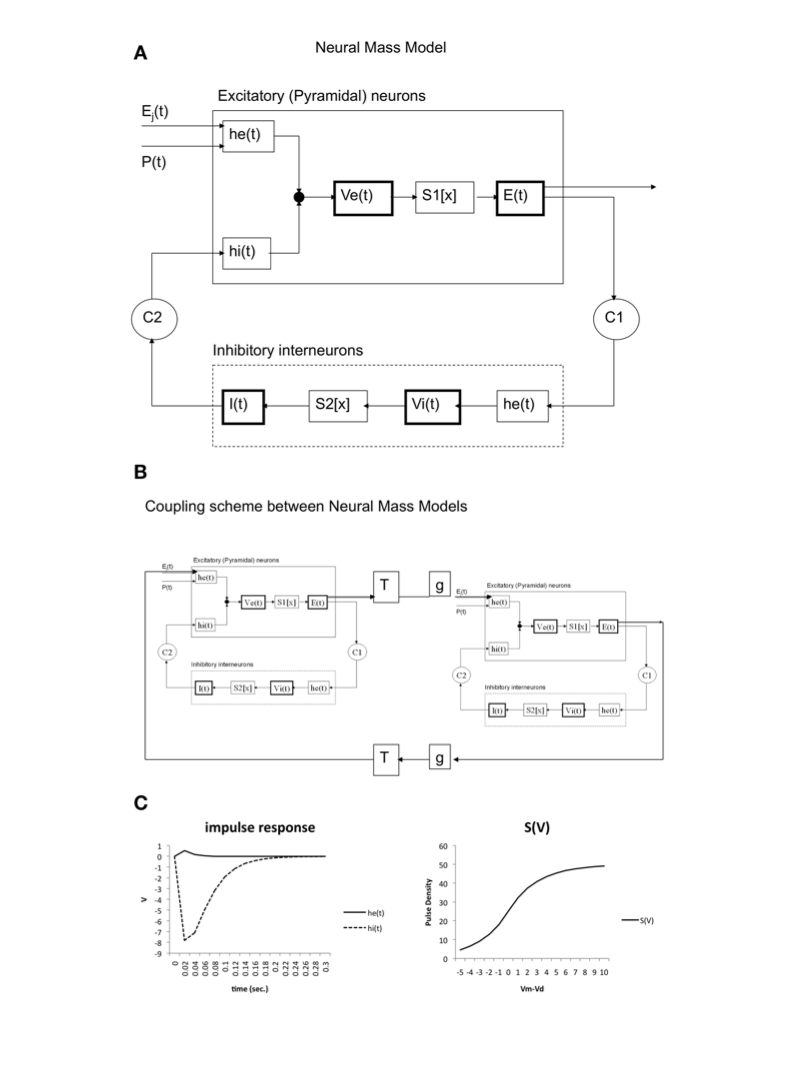


D


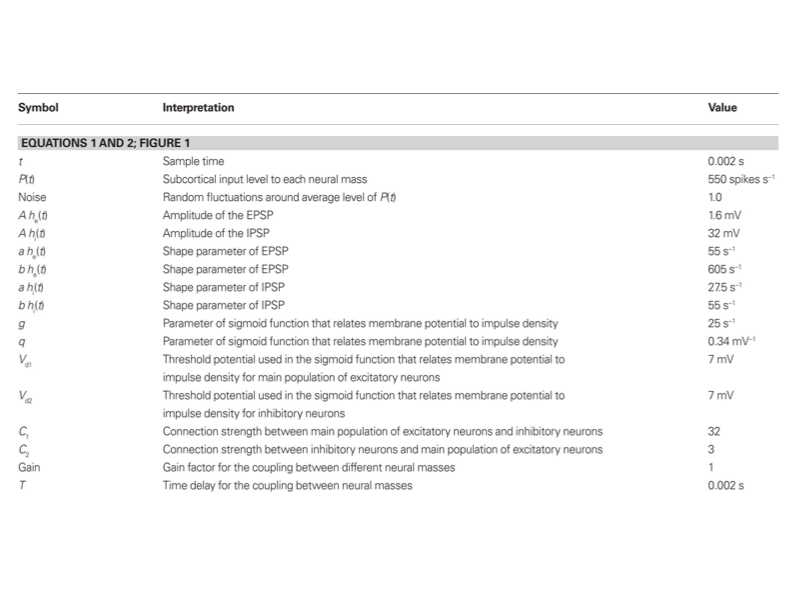

Supplement: S1 Fig — (A) Schematic presentation of single neural mass model. The upper rectangle represents a mass of excitatory neurons, the lower rectangle a mass of inhibitory neurons. The state of each mass is modeled by an average membrane potential [Ve(t) and Vi(t)] and a pulse density [E(t) and I(t)]. Membrane potentials are converted to pulse densities by sigmoid functions S1[x] and S2[x]. Pulse densities are converted to membrane potentials by impulse responses he(t) and hi(t). C1 and C2 are coupling strengths between the two populations. P(t) and Ej(t) are pulse densities coming from thalamic sources or other cortical areas respectively. (B) Coupling of two neural masses. Two masses are coupled via excitatory connections. These are characterized by a fixed delay T and a strength g. (C) Essential functions of the model. The upper left panel shows the excitatory [he(t)] and inhibitory [hi(t)] impulse responses of Eq. 1. The upper right shows the sigmoid function relating average membrane potential to spike density (Eq. 2). (D) Overview of neural mass model parameters. (DOC) [file pcbi.1005707.s001.doc]

A


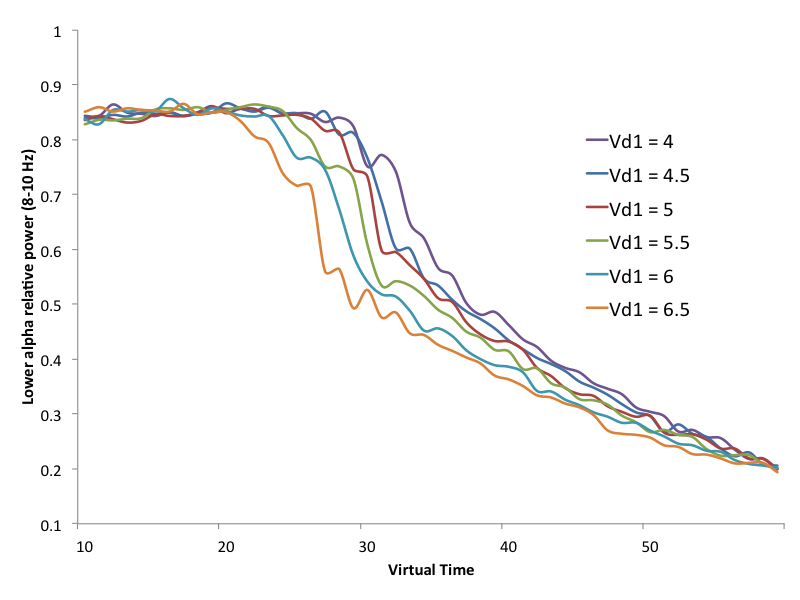


B


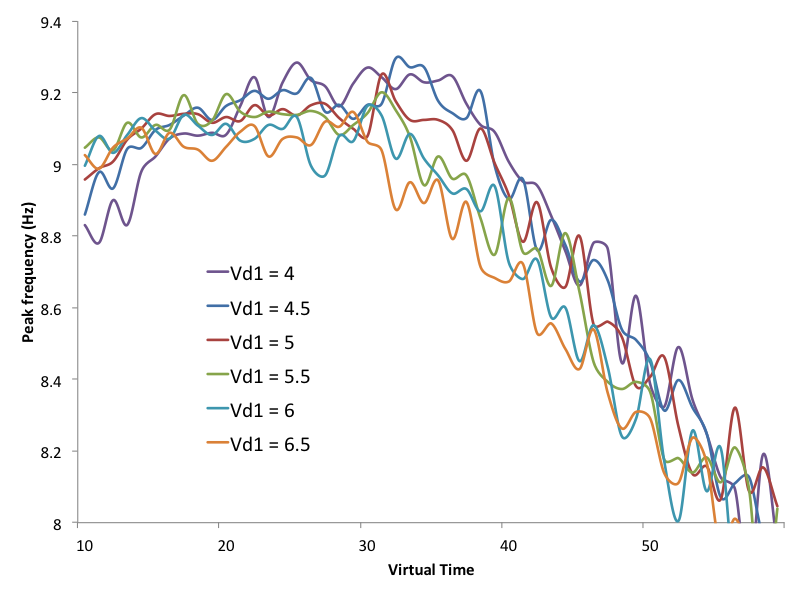


C


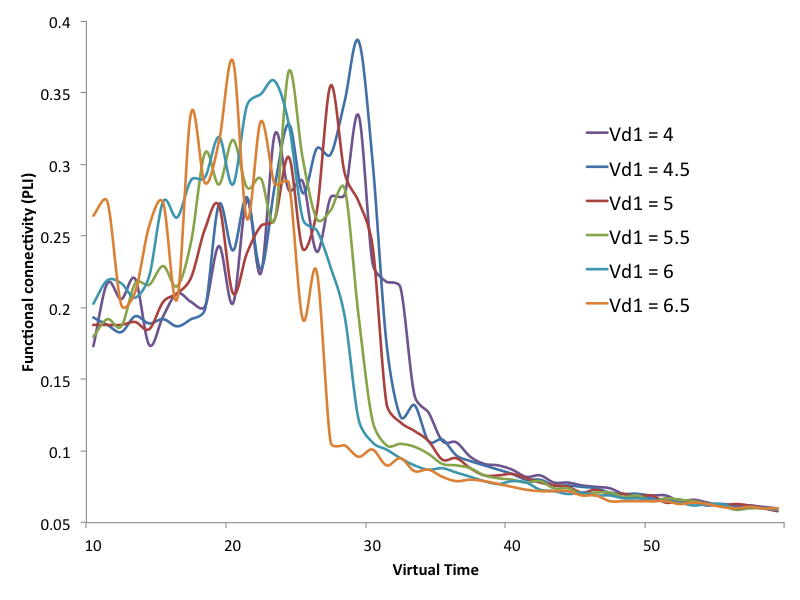


D


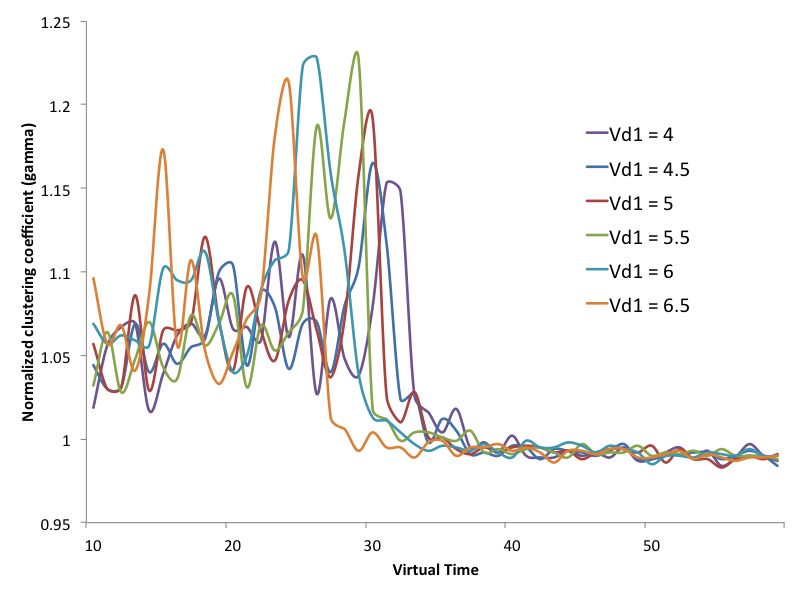


E


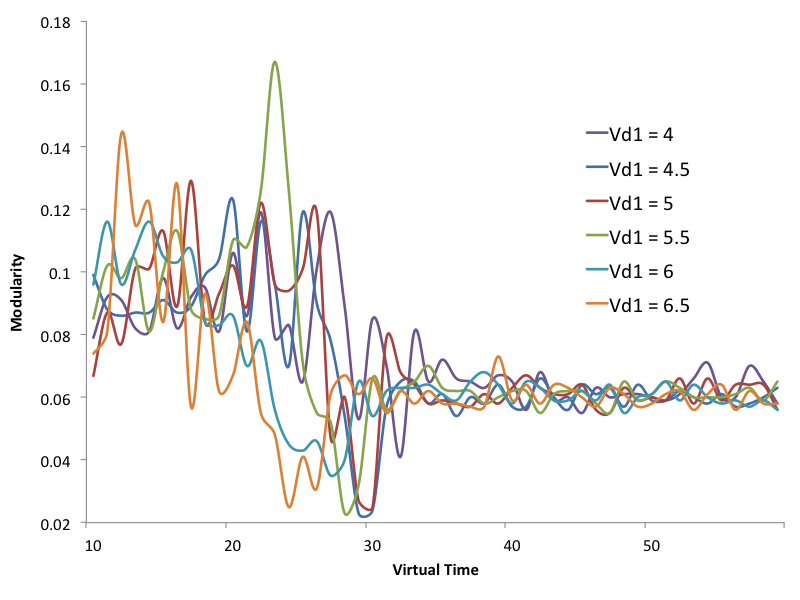


F


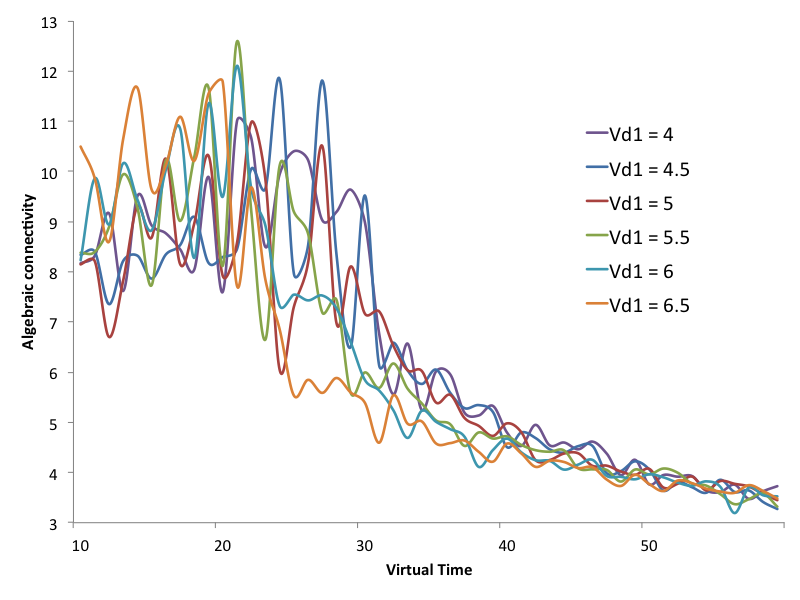


G


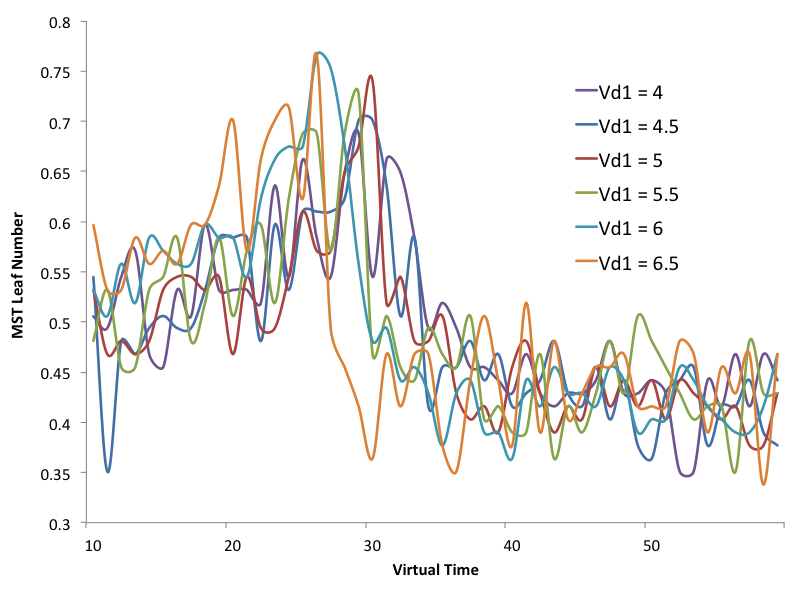

Supplement: S2 Fig — (A) The effect of Vd level on lower alpha relative power during the ‘stimulation of excitatory neurons’ scenario. (B) The effect of Vd level on peak frequency during the Stimulation of excitatory neurons scenario. (C) The effect of varying Vd levels on PLI during the 'Stimulation of excitatory neurons' strategy. A lower Vd1 seems related to a longer lasting normal PLI level. (D) The effect of Vd level on the normalized clustering coefficient (gamma, a measure of local connectivity) during the 'Stimulation of exctiatory neurons' scenario. (E) The effect of Vd level on algebraic connectivity (robustness) during the 'Stimulation of excitatory neurons' scenario. (F) The effect of Vd level on modularity (subnetwork presence) during the 'Stimulation of excitatory neurons' scenario. (G) The effect of Vd level on MST Leaf Number (network hub presence) during the 'Stimulation of excitatory neurons' scenario. (DOC) [file pcbi.1005707.s002.doc]

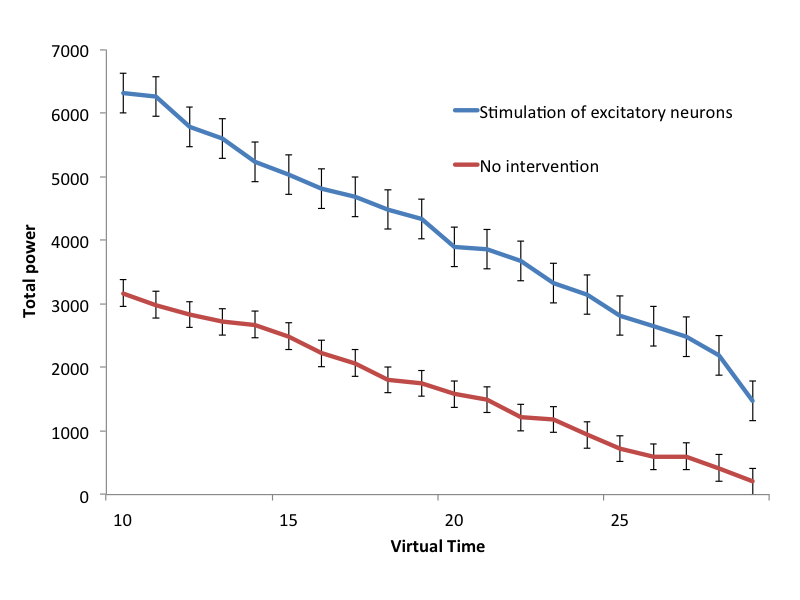

Supplement: S3 Fig — (TIF) [file pcbi.1005707.s003.tif]
