# Supplementary material for: Altering neuronal excitability to preserve network connectivity in a computational model of Alzheimer's disease
Source: PLoS Comput Biol. 2017 Sep 22;13(9):e1005707. doi: 10.1371/journal.pcbi.1005707 (PMC5627940; doi:10.1371/journal.pcbi.1005707)
Supplement: S1 Text — (DOC) [file pcbi.1005707.s004.doc]

**Supporting Information**

1) The Neural Mass Model

We used a model of interconnected neural masses, where each neural mass represents a large population of connected excitatory and inhibitory neurons generating an EEG or MEG like signal. The model was described in Ponten et al. and Stam et al. [1,2]. The basic unit of the model is a neural mass model (NM) of the alpha rhythm [3,4]. As previously described, this model considers the average activity in relatively large groups of interacting excitatory and inhibitory neurons. Spatial effects are ignored in this model; we introduced topological effects by coupling several NMs together. The excitatory and inhibitory populations of each NM are characterized by their average membrane potentials *V*_e_(*t*) and *V*_i_(*t*), and by their pulse densities, i.e., the proportion of cells firing per unit time *E*(*t*) and *I*(*t*). Static non-linear functions *S*_E_(*x*) and *S*_I_(*x*) relate the potentials *V*_e_(*t*) and *V*_i_(*t*) to the corresponding pulse densities *E*(*t*) and *I*(*t*). The excitatory post-synaptic potential (EPSP) and inhibitory post-synaptic potential (IPSP) are modeled by the impulse responses *h*_e_(*t*) and *h*_i_(*t*). The constants *C*_1_ and *C*_2_ describe the coupling from excitatory to inhibitory and from inhibitory to excitatory populations respectively. *P*(*t*) is the pulse density of an input signal to the excitatory population. Following Zetterberg et al. [4] the following impulse responses were used:


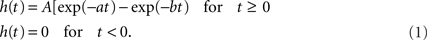


For *h*_e_(*t*) the parameter values were: *A* = 1.6 mV, *a* = 55 s^−1^, *b* = 605 s^−1^. For *h*_i_(*t*) the parameter values were: *A* = 32 mV, *a* = 27.5 s^−1^, *b* = 55 s^−1^. The sigmoid function relating the average membrane potential, *V*_m_, to the impulse density was also taken from Zetterberg et al. [4]:


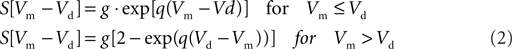


Here the parameter values used were: *q* = 0.34 mV^−1^, *V*_d_ = 7 mV, *g* = 25 s^−1^. For the coupling constants we used *C*_1_ = 32 and *C*_2_ = 3 [3].

A schematic representation is shown in figure S1 Fig1 (panel A). A schematic illustration of the coupling between two NMs is shown in figure S1 Fig 1 (panel B). The impulse response and sigmoid functions are shown in figure S1 Fig1 (panel C). All model parameters are summarized in figure S1 Fig 2.

The final model consisted of 78 of the neural masses as described above, which were coupled together based on the structural DTI network results from Gong et al. [5]. Coupling between two NMs, if present, was always reciprocal, and excitatory. The output *E*(*t*) of the main excitatory neurons of one NM was used as the input for the impulse response *h*_e_(*t*) of the excitatory neurons of the second NM; the output *E*(*t*) of the second module was coupled to the impulse response *h*_e_(*t*) of the excitatory neurons of the first NM. Following Ursino et al. [6] we used a time delay (*T* × sample time, with *n* an integer, 0 < *T* < 21) and a gain factor. In the present study, *n* and gain were set to 1 for all connections. For the present study the model was extended in order to be able to deal with activity-dependent degeneration of connection strength between multiple coupled NMs. Coupling strength between neural masses was initially set at the same level for all connections; S=1.5 [7].

The average membrane potential of the excitatory neurons *V*_e_(*t*) of each of the NMs separately was the multichannel output. The sample frequency was 500 Hz. In the present study each run consisted of 4096 samples (±8 s). Amount of data selected for analysis was based on previous studies and literature on reproducibility of graph theoretical results [8,9]. The adjacency matrix at the end of each run was subjected to topographical analysis. Figure S2 Fig 2 gives an overview of model parameters and initial settings. These parameters go back to a large number of studies with this lumped model, and ultimately to the original model of Lopes da Silva [3].

Activity dependent degeneration (ADD) was realized by lowering the ‘synaptic’ coupling strength as a function of the spike density of the main excitatory neurons [7]. For each neural mass the spike density of the main excitatory population is stored in a memory buffer that contains the firing rates of the last 20 steps in the model. Step size depends on the sample frequency. With each new iteration, the highest spike density value of the last 20 sample steps is determined and designated as ‘maxAct’. From maxAct a loss is determined according to the following relation:

$loss=\exp^{-0.01\max Act}$ (3)

Since maxAct is non-negative, loss will be a number between 0 and 1. Next, the coupling values C1 (connections between main excitatory population and inhibitory population), C2 (connections between inhibitory population and main excitatory population), Pt (thalamic input to main excitatory population) and S (*structural* coupling strength between neural masses) are all multiplied by loss to obtain their new lower values.

Model output is generated for >50 runs of the degeneration algorithm, to simulate a progressive neurodegenerative process over time. Therefore, the X-axis variable has been defined as ‘virtual time’. Note that there is no defined relation to real time; this parameter should not be interpreted as hours, days or otherwise.

**References**

1. Ponten SC, Daffertshofer a., Hillebrand a., Stam CJ. The relationship between structural and functional connectivity: graph theoretical analysis of an EEG neural mass model. Neuroimage 2010;52(3):985–94.

2. Stam CJ. Characterization of anatomical and functional connectivity in the brain: a complex networks perspective. Int J Psychophysiol 2010;77(3):186–94.

3. da Silva FH, Hoeks A, Smits H, Zetterberg LH. Model of brain rhythmic activity. Biol Cybern. Springer; 1974;15(1):27–37.

4. Zetterberg LH, Kristiansson L, Mossberg K. Performance of a model for a local neuron population. Biol Cybern. Springer; 1978;31(1):15–26.

5. Gong G, He Y, Concha L, Lebel C, Gross DW, Evans AC, et al. Mapping anatomical connectivity patterns of human cerebral cortex using in vivo diffusion tensor imaging tractography. Cereb Cortex 2009;19(3):524–36.

6. Ursino M, Zavaglia M, Astolfi L, Babiloni F. Use of a neural mass model for the analysis of effective connectivity among cortical regions based on high resolution EEG recordings. Biol Cybern 2007;96(3):351–65.

7. de Haan W, Mott K, van Straaten ECW, Scheltens P, Stam CJ. Activity Dependent Degeneration Explains Hub Vulnerability in Alzheimer’s Disease. PLoS Comput Biol. 2012;8(8).

8. Deuker L, Bullmore ET, Smith M, Christensen S, Nathan PJ, Rockstroh B, et al. Reproducibility of graph metrics of human brain functional networks. Neuroimage 2009;47(4):1460–8.

9. Whitlow CT, Casanova R, Maldjian JA. Effect of resting-state functional MR imaging duration on stability of graph theory metrics of brain network connectivity. Radiology 2011;259(2):516.
