# Supplementary material for: Altering neuronal excitability to preserve network connectivity in a computational model of Alzheimer's disease
Source: PLoS Comput Biol. 2017 Sep 22;13(9):e1005707. doi: 10.1371/journal.pcbi.1005707 (PMC5627940; doi:10.1371/journal.pcbi.1005707)
Supplement: S2 Text — (DOC) [file pcbi.1005707.s005.doc]

**Supporting Information**

**2) Membrane threshold potential (Vd) selection**

To examine network behavior for various excitation/inhibition strengths within the different strategies, and to pick a representative Vd threshold for further analysis, we have performed the simulation for each strategy with 6 different Vd values. As mentioned in the method section, these span the entire realistic range, from Vd=4 up to Vd=10. Vd values outside this range produce very erratic network behavior (not shown) in most strategies, such as an abrupt complete breakdown of activity (in the case of inhibition) or uncontrolled hyperactivity (excitation). The descriptive figures S2 Fig 1 - 7 display this analysis for the most successful, ‘Stimulation of excitatory neurons’ scenario. For each outcome measure the effect of Vd alteration is shown.

As can be judged from the figures, the timecourse of the measures using different Vd values is fairly similar, but Vd levels further away from the normal situation (Vd=7) in general appear to lead to longer preservation of network integrity. For this specific strategy, we chose the Vd1=5 variant to be included in the further analysis. Note that Vd1 is altered the affect excitatory neurons, and Vd2 for inhibitory neurons.
