# Supplementary material for: Altering neuronal excitability to preserve network connectivity in a computational model of Alzheimer's disease
Source: PLoS Comput Biol. 2017 Sep 22;13(9):e1005707. doi: 10.1371/journal.pcbi.1005707 (PMC5627940; doi:10.1371/journal.pcbi.1005707)
Supplement: S3 Text — (DOC) [file pcbi.1005707.s006.doc]

**Supporting Information**

**3)** **Single neural mass activity comparison between the ‘Stimulation of excitatory neurons’ scenario and the ‘No intervention’ state.**

To assess whether the relative success ‘Stimulation of excitatory neurons’ strategy was based on a net inhibitory effect in the activity within a single neural mass, we compared total power values for this strategy and the ‘no intervention’ condition. Figure S3 Fig 1 shows that this is not the case: the total activity level of the neural mass increases. Therefore, since all neural masses are identical (except for their connectivity) the explanation of how stimulation of a hyperactive network can be relatively beneficial may lie in the network topology itself (see also the Discussion)
