# Supplementary material for: Altering neuronal excitability to preserve network connectivity in a computational model of Alzheimer's disease
Source: PLoS Comput Biol. 2017 Sep 22;13(9):e1005707. doi: 10.1371/journal.pcbi.1005707 (PMC5627940; doi:10.1371/journal.pcbi.1005707)
Supplement: S4 Text — (DOCX) [file pcbi.1005707.s007.docx]

**Supporting Information**

**4) Graph measure definition**

**Local connectivity: the normalized clustering coefficient (gamma)**

The clustering index *Ci* of a vertex *i* generally represents the likelihood that other

vertices *j* that are connected to the vertex *i* will also be connected to each other. This notion can be adopted for use with weighted graphs in various ways (Boccaletti *et al.*, 2006). Here we propose a simple definition, closely related to the proposal of Onnela *et al.* (2005), which only requires symmetry (*wij* = *wji*) and that 0 ≤ *wij* ≤ 1 holds. Indeed, both conditions are readily fulfilled when using PLI as weight definition. The (weighted) clustering index of vertex *i* is then defined as:


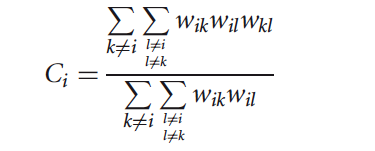


Notice that in all sums terms with *k* = *i*, *l* = *i*, or *k* = *l* are skipped. In the special case in which *wij* equals either 0 or 1, this definition is equivalent to the classical definition for unweighted graphs (Watts and Strogatz, 1998). For isolated vertices, i.e. vertices that do not have any connections, all weights *wij* vanish, and the clustering index is defined as Ci=0 (Newman, 2003). The mean clustering coefficient of the entire network can be determined via as:


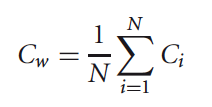


By definition, *Cw*  depends on edge weights and network structure but also on network size. In order to obtain measures that are independent of network size, the mean edge weights


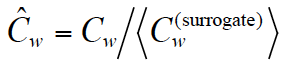


were computed, in which
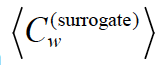
denotes weighted clustering coefficient and path length averaged over an ensemble of 100 surrogate random networks that were derived from the original networks by randomly reshuffling the edge weights.

**Community detection: the modularity index**

To describe modularity in the whole-brain network we used a modification of the approach by Guimera and Nunes Amaral (Guimera` and Amaral, 2005; Newman and Girvan, 2004), adapted for weighted networks and identical to Stam et al. (Stam, 2010):


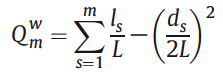


where *m* is the number of modules, *ls* the sum of the weights of all links in module *s*, L is the total sum of all weights in the network, *ds* is the sum of the strength of all vertices in module *s*. In short, the relation between intra- and intermodular connections determines the strength of each module. For any given network partition, this measure describes the strength of the total modularity by summing the relative strength of all the modules in the network. A strongly modular network has modularity value close to 1, and in a network without modular organization it will approach 0. Finding the optimal modular organization in a network is a computationally intensive problem. One of the most effective methods to date is *simulated annealing* (Guimerà *et al.*, 2004; Guimerà and Amaral, 2005). This method was used to find the optimal way to divide the network into modules: initially, each of the *N* nodes was randomly assigned to one of *m* possible clusters, where *m* was taken as the square of *N*. At each step, one of the nodes was chosen at random, and assigned a different randomly chosen module number from the interval [1,*N*]. Modularity was calculated before and after this. The cost *C* was defined as
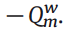
. The new partitioning was preserved with probability p:

**
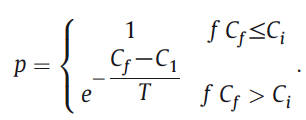
**

Here, *Cf* is final cost and *Ci* is initial cost. The temperature *T* was 1 initially, and was lowered once every 100 steps as follows: *Tnew* = 0.995 *Told*. In total, the simulated annealing algorithm was run for 10^6^ steps. The partition with the strongest modular organization was identified separately for each epoch of every person for all the different frequency bands, and subjected to further graph analysis.

**Robustness: algebraic connectivity (graph spectral analysis)**

Graph spectral measures are derived from the adjacency or *Laplacian matrix Q.* This is done by subtracting the adjacency matrix from the *degree matrix* Δ (Q=Δ-A), which is the diagonal matrix with the nodal degrees (equal to the rowsum of the adjacency matrix). The Laplacian matrix can be regarded as a way to combine both connectivity and degree information (all relevant information) in the same matrix. Both the adjacency and Laplacian matrix can be written in terms of their eigenvectors and corresponding eigenvalues, e.g. A=XΛX^T^, where the matrix X consists of all eigenvectors in columns and the diagonal matrix Λ contains the corresponding eigenvalues. The spectral information (X and Λ) thus contains the same information as the topology, or adjacency matrix (Van Mieghem, 2011).

The spectrum of a graph can be regarded as a unique ‘fingerprint’. Especially the different eigenvalues contain precise information about network properties, and can be used to quantitatively classify network topologies.

The algebraic connectivity measures how difficult it is to tear a network apart. If the network is fully connected, the algebraic connectivity is greater than 0. The magnitude of the algebraic connectivity can also be regarded as a measure for network ‘robustness’. The algebraic connectivity is equal to the second-smallest eigenvalue of the Laplacian matrix (Fiedler, 1973; Mohar, 1991; Van Mieghem, 2011).

**Hub Presence: leaf number (minimum spanning tree analysis)**

The minimum spanning tree (MST) is a sub-network of the original weighted network that connects all nodes in the network without forming loops and has the minimum total weight of all possible spanning trees. MSTs are constructed based on the weighted networks with Kruskal's algorithm (Kruskal, 1956). In our case we start the algorithm with the largest link weights since we are interested in the strongest connections in the network. This algorithm first orders the weights of all links in a descending order and starts the construction of the minimum spanning tree with the largest link weight and adds the following largest link weight until all nodes N are connected in an acyclic sub-network that consists of M = N − 1 links, i.e. a fixed density. When in the process, addition of a link forms a loop, this link is ignored. After construction of the MST, all link weights are assigned a value of one.

There are two extreme tree topologies; path- and star-like configurations. Path-like configurations consist of nodes that are all linked to two other nodes with the exception of the nodes at either end of the path. Nodes with only one link in a tree are referred to as ‘leaf’ nodes (or leaves) and the number of those nodes in a tree is the leaf number. Thus a path has a leaf number of two. In contrast, star-like configurations consist of a central node connected to all other nodes with only one link. Thus, a star consisting of *N* nodes has a leaf number of *N* − 1.

The leaf number has a lower bound of 2 and upper bound of *N* − 1. The leaf number presents an upper bound to the diameter of the MST, which is the largest distance between any two nodes of the tree. Therefore, a low leaf number represents a path-like network without notable hub structure, whereas a high number represent a star-like configuration network with hubs. Here, we present the leaf numer as a fraction of the total number of nodes in the tree, leading to a value between 0 and 1 (see also [73]).

**Structural connectivity – normalized node strength**

The ‘normalized node strength’ is the ratio of the structural degree of a node after activity dependent damage over its original degree. This measure was used to track structural connectivity loss due to the damage algorithm, and was also used in [18].
